# Nrf2 inhibition increases sensitivity to chemotherapy of colorectal cancer by promoting ferroptosis and pyroptosis

**DOI:** 10.1038/s41598-023-41490-x

**Published:** 2023-09-01

**Authors:** Yongzhou Huang, Wenchang Yang, Lei Yang, Tao Wang, Chengguo Li, Jiaxian Yu, Peng Zhang, Yuping Yin, Ruidong Li, Kaixiong Tao

**Affiliations:** 1 Department of Gastrointestinal Surgery, Union Hospital, Tongji Medical College, Huazhong University of Science and Technology, No. 1277 Jiefang Avenue, Wuhan, 430022 Hubei Province People’s Republic of China; 2 Department of General Surgery, First Affiliated Hospital of Shihezi University, Shihezi, Xinjiang, 832008 People’s Republic of China

**Keywords:** Cancer, Cell biology, Chemical biology, Drug discovery

## Abstract

Oxaliplatin is widely used in chemotherapy for colorectal cancer (CRC), but its sensitivity has become a major obstacle to limiting efficacy. Many literatures reported that Nrf2 activation promoted tumor chemoresistance. In this study, we explored the role and mechanism of Nrf2 inhibition in oxaliplatin-based chemosensitivity of CRC. In vitro experiments, we applied 4-octyl itaconate (4-OI) to activate Nrf2, and used lentivirus to knock down Nrf2 in CRC cell lines. By measuring cell viability, colony formation, apoptosis, reactive oxygen species production, and western blot, we found that oxaliplatin and lobaplatin suppressed the growth of HCT-116 and LOVO cells in a dose-dependent manner, and promoted the expression of Nrf2. 4-OI, an Nrf2 activator, reduced the sensibility of CRC cells to oxaliplatin and lobaplatin, while the knockdown of Nrf2 promoted the sensibility of CRC cells to oxaliplatin and lobaplatin. Through the public databases, we found that the expression of GPX4 in normal tissues was lower compared with cancer tissues in CRC, and the high GPX4 expression predicted a poor prognosis. Meanwhile, we found that oxaliplatin reduced the expression of GPX4 in vitro. The knockdown of Nrf2 enhanced the effects of oxaliplatin to reduce the expression of GPX4 and GSH content, and increase the MDA content, which enhanced oxaliplatin-induced ferroptosis. Subsequently, we found that oxaliplatin promoted the expression of GSDME-N, and induced LDH, IL-1β, and TNF-a release, and the knockdown of Nrf2 aggravated the occurrence of GSMDE-mediated pyroptosis. Finally, we found that the knockdown of Nrf2 enhanced the inhibition of oxaliplatin on HCT116 xenograft tumor growth in vivo. Thus, our study showed that Nrf2 inhibition improved sensitivity to oxaliplatin of CRC cells by promoting ferroptosis and pyroptosis, which provided a new target for overcoming chemoresistance in CRC.

## Introduction

Colorectal cancer (CRC) is the second most deadly cancer among adults in the world ^1^. Currently, the oxaliplatin-based FOLFOX and CAPOX schemes are considered as the preferred chemotherapeutic scheme for advanced CRC after complete resection^2,3^. Numerous studies have revealed that oxaliplatin significantly improved the prognosis of advanced CRC patients, but some studies also showed approximately 50% of advanced CRC patients did not benefit from it^4,5^. Low sensitivity to chemotherapy treatment is an important cause for the poor effect^6^. Therefore, it is important to develop new targets to improve the sensitivity to chemotherapy, in order to further improve the prognosis of CRC patients.

Nuclear factor erythroid 2-related factor 2 (Nrf2) is a classic anti-inflammatory and antioxidant molecule, which can suppress ROS production and improve the ability of cells to adapt to the external environment^7,8^. Studies have revealed that constitutive activation of the Nrf2 in many cancers contributes to cancer cell proliferation, prevents apoptosis, enhances the self-renewal capacity, and more importantly, can reduce the chemical sensitivity of cancer cells^9–11^. Nrf2 axis activation inhibits ferroptosis through the abolishment of lipid oxidation^12,13^. Numerous researches have shown that promoting ferroptosis tumor cells plays an excellent anti-tumor effect in many malignant tumors^14,15^. Furthermore, Nrf2 activation inhibits ROS production, which further attenuates caspase3 activation and ultimately attenuates GSDME-mediated pyroptosis^16–18^. Recently, the role of GSDME has attracted more and more attention, and many studies showed its excellent anti-tumor effects^19–21^. Zheng et al.^22^ have reported that metformin amplified NF-κB signaling to promote caspase3/GSDME-mediated cancer cell pyroptosis. An et al.^23^ revealed that tetraarsenic hexoxide promoted triple-negative breast cancer cells pyroptosis by activating caspase-3/GSDME. Therefore, we envisage that suppressing the Nrf2 pathway could be a potential target in promoting ferroptosis and pyroptosis, which is expected to improve the sensitivity to chemotherapy of CRC.

Currently, numerous experiments have shown that itaconate and its derivative 4-octyl itaconate (4-OI) exert anti-inflammatory and antioxidant effects by activating Nrf2. Meanwhile, Mill et al.^24^ have revealed that itaconate alkylates cysteine residues 151, 257, 288, 273 and 297 on the protein KEAP1 to activate Nrf2. Our previous experiments also showed that 4-OI was an excellent Nrf2 activator. Therefore, we chose the 4-OI as an Nrf2 activator. Here, our study showed that the knockdown of Nrf2 improves sensitivity to the oxaliplatin and lobaplatin of CRC cells. Significantly, we demonstrated that the knockdown of Nrf2 increases sensitivity to chemotherapy of CRC by promoting ferroptosis and pyroptosis.

## Methods

### Cell lines and drugs

The human CRC cell lines HCT116 and LOVO were obtained from the American Type Culture Collection (Manassas, VA, USA). Cells were cultured in Dulbecco’s modified Eagle’s medium (DMEM) medium supplemented with 10% fetal bovine serum (FBS, Gibco) in a 5% CO_2_ humidified incubator at 37 °C. 4-OI, ML385, Dimethyl fumarate (DMF), oxaliplatin, and lobaplatin were obtained from Med Chem Express (New Jersey, USA) and dissolved in dimethyl sulfoxide (DMSO) at − 80 °C.

### Cell viability assay

The cell viability was evaluated by the 3-(4,5-dimethylthiazol-2-yl)-5-(3-carboxymethoxyphenyl)-2-(4-sulfophenyl)–2H-tetrazolium (MTS) kit (Promega, Madison, USA). The cells of the logarithmic growth phase were seeded in 96-wells plates at a density of 2000 cells per well. HCT116 and LOVO cells were dealt with diverse concentrations of oxaliplatin and lobaplatin. After 48 h of treatment, the liquid in the 96 well plates was sucked out, then an equal volume culture medium was added. Finally, 10µL MTS solution was added to each well and incubated at 37 °C for 40 min. The absorbance of each well was detected by a microplate reader at 490 nm.

### Clonogenic assays

HCT116 and LOVO cells (1000 cells/well) were seeded in 6-well plates and were treated with oxaliplatin and lobaplatin for 48 h. After two weeks, colonies were fixed by 4% paraformaldehyde and crystal violet was used to stain the cells. All experiments were conducted in triplicate.

### Lentivirus transduction

Control short hairpin RNAs and lentiviruses targeting Nrf2 were bought from Genechem (Shanghai, China) (http://www.genechem.com.cn/service/index.php?ac=gene&at=vector_search&keyword=GV493). The sequences were as follows: RNAi: GGGCAACTGTGGTCCACATTT. The ID is Nfe2l2-RNAi (67,328-1). The knockdown efficiency was examined using western blots.

### Apoptosis analysis

The apoptosis analysis was detected through the Annexin V/7-AAD apoptosis kit (BioLegend, California, USA). Normal and Nrf2 knockdown cells were seeded in 6-well plates and dealt with oxaliplatin, lobaplatin, dimethyl fumarate (DMF) or 4-OI for 48 h. Then, cells were harvested and washed by precooled phosphate-buffered saline (PBS) for three times. Annexin V binding buffer (100 µL) suspended the cells. APC–Annexin V (5 µL) and 7-AAD (10 µL) were added and vortexed. Finally, the cells were incubated for 15 min before analyzing through flow cytometry.

### Detection of reactive oxygen species (ROS) level

Dihydroethidium (Beyotime, Shanghai, China) was used for detecting the ROS levels according to the manufacturer’s instructions. In brief, cells were seeded in 6-well plates and dealt with Oxaliplatin, Lobaplatin, and 4-OI for 48 h. Subsequently, cells were harvested and washed by PBS for two times. The cells were labeled with dihydroethidium for 30 min in the dark. Finally, the mean fluorescence intensity was tested by flow cytometry. The ROS activities of frozen tumor sections were detected by dihydroethidium (DHE).

### Measurement of malondialdehyde (MDA)

Cell malondialdehyde (MDA) assay kit (Nanjing Jiancheng Institute of Biotechnology, Nanjing, China) was used for measuring cellular MDA contents according to the manufacturer’s instructions.

### Measurement of glutathione

Glutathione was measured using the glutathione kit (Nanjing Jiancheng Institute of Biotechnology, Nanjing, China) according to the manufacturer’s instructions.

### Lactate dehydrogenase (LDH) release assay

Cells were treated as described above, then the supernatant was collected. The LDH release was evaluated using an LDH assay kit (Beyotime, Shanghai, China) according to the manufacturer’s instructions.

### Enzyme-linked immunosorbent (ELISA) assay

The levels of tumor necrosis factor-α (TNF-a) and interleukin-1β (IL-1β) were determined by ELISA kits (DAKEWE Bioengineering, Beijing, China) following the manufacturer’s instructions.

### Bioinformatics analysis

The mRNA expression of colon cancer patients was obtained from the UCSC Xena project (http://xena.ucsc.edu). The prognosis analysis was downloaded from OncoLnc (http://www.oncolnc.org/).

### Immunohistochemistry (IHC)

Tumor tissues were fixed, dehydrated, embedded in paraffin, and serially sectioned at a thickness of 4 μm. Then the sections were incubated with primary and secondary antibodies. Briefly, the paraffin‐embedded tissue was dried, dewaxed, and rehydrated. Then the sections were rinsed phosphate with buffered saline solution (PBS, Beyotime, Shanghai, China) and blocked with bovine serum albumin (Beyotime, Shanghai, China). The sections were incubated with Ki-67 monoclonal antibody (34,330, Cell Signaling Technology, MA, USA) and cle-caspase3 (9664, Cell Signaling Technology, MA, USA) at 4 °C overnight. The sections were incubated with a secondary antibody the next day for 30 min at 37 °C. The diaminobenzene was used as the chromogen, and hematoxylin was used as the nuclear counterstain. The IHC scores were obtained by multiplying the percentage of positive tumor cells and the intensity of staining. The value of positive tumor cells was defined as 0 (no staining), 1 (0%–25%), 2 (25%–50%) and 3 (> 50%). The value of intensity of staining was defined as 0, 1, 2 and 3^25^.

### Western blot analysis

Protein was extracted from cells with RIPA lysis buffer and protease inhibitor. The protein concentrations were measured by BCA protein assay (Beyotime, Shanghai, China). Proteins were separated by 10% Sodium dodecyl sulphate–polyacrylamide gel electrophoresis (SDS-PAGE) and were transferred to a polyvinylidene fluoride (PVDF) membranes (0.22 µm or 0.45 µm). The membranes were blocked for 1 h and then incubated with primary antibodies for 16 h. The primary antibodies included anti-GAPDH (1:3000; Proteintech), anti-Lamin B1(1:3000; Proteintech), anti-GPX4 (1:1000; Abcam), anti-Nrf2 (1:1000; Cell Signaling Technology), anti-HO-1 (1:1000; Proteintech), anti-GSDME (1:1000; Abcam). The next day, the membranes were washed with TBST for three times. Then the membranes were incubated with secondary antibodies including HRP-conjugated goat anti-rabbit (1:3000; Proteintech). Finally, the membrane was imaged with ECL reagents (Cell Signaling Technology, MA, USA) by iBright CL1000 imaging system. In order to save antibodies and reduce experimental operation time, we ran the same molecules from different treatment groups together. We provided all replicates in the Supplementary Information file.

### Animal studies

Male 5-week-old Balb/c nude mice were purchased from HUAFUKANG Bioscience (Beijing, China). The animal studies were conducted in compliance with the Animal Research: Reporting In Vivo Experiments (ARRIVE) guidelines with the approval of the Institutional Animal Care and Use Committee at Tongji Medical College, Huazhong University of Science and Technology ([2022] IACUC Number: 2988). Also, the studies were carried out according to the Guide for the Care and Use of Laboratory Animals, and in strict accordance with the People’s Republic of China Legislation Regarding the Use and Care of Laboratory Animals. HCT116 shscramble or shNrf2 cells (2 × 10^6^) in 100μL PBS were subcutaneously injected into the right flank of mice to establish the nude mice tumor model. Two weeks later, the mice were randomly divided into four groups: control, shNrf2, Oxaliplatin, and shNrf2 plus Oxaliplatin groups (n = 6). Oxaliplatin (5 mg/kg) was administered via intraperitoneal injection every 2d. The tumor volume was calculated using a formula [(length × width × width)/2]. The tumor volume was measured every 2d. After treatment for 20d, mice were anesthetized with isoflurane before being euthanized. The transplanted tumors were excised for the subsequent experiments.

### Statistical analysis

The results were expressed by the mean ± SD of three independent experiments. The differences between two groups or multiple groups were determined through a *t*-test and one-way analysis of variance. SPSS 25.0 and GraphPad Prism 8.0 software were used for statistical analysis. A difference of *p* < 0.05 was regarded as statistical significance.

### Ethical approval

The animal studies were conducted in compliance with the Animal Research: Reporting In Vivo Experiments (ARRIVE) guidelines with the approval of the Institutional Animal Care and Use Committee at Tongji Medical College, Huazhong University of Science and Technology ([2022] IACUC Number: 2988). Also, the studies were carried out according to the Guide for the Care and Use of Laboratory Animals, and in strict accordance with the People’s Republic of China Legislation Regarding the Use and Care of Laboratory Animals.

## Results

### Oxaliplatin and lobaplatin had cytotoxic effects on CRC cells and promoted the expression of Nrf2.

**Figure 1:**
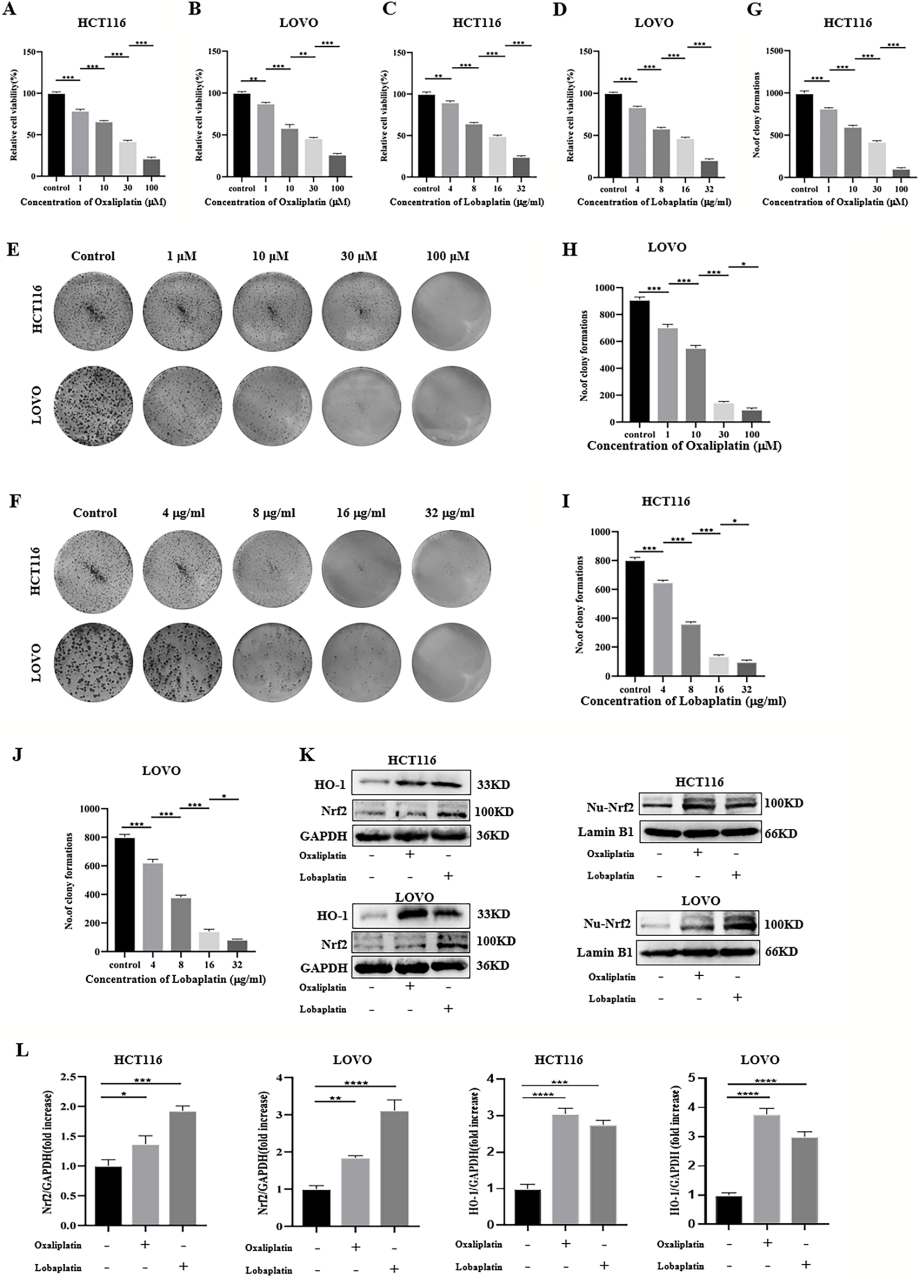
Effects of oxaliplatin and lobaplatin on the growth of HCT‐116 and LOVO cells. (**A**, **B**) Oxaliplatin reduced CRC cells viability by the MTS assay. (**C**, **D**) Lobaplatin reduced CRC cells viability by the MTS assay. (**E**–**J**) Effects of oxaliplatin and lobaplatin on CRC cells colony formation. (**K**, **L**) Oxaliplatin and lobaplatin promoted the expression of Nrf2/HO-1 in overall protein and nuclear protein. Data are shown as the mean ± SD, *p < 0.05; **p < 0.01; ***p < 0.001.

To assess the cytotoxic effects of oxaliplatin and lobaplatin on CRC cells, Two CRC cell lines (HCT116 and LOVO) were treated with different concentrations of oxaliplatin (0μM, 1 μM, 10 μM, 30 μM, 100 μM) or lobaplatin (0 μg/ml, 4 μg/ml, 8 μg/ml, 16 μg/ml, 32 μg/ml) for 48 h. The cell viability was detected by the MTS assay. We found that oxaliplatin and lobaplatin suppressed the growth of HCT-116 and LOVO cells in a dose-dependent manner (Fig. 1A–D). On the basis of these observations, subsequently, we evaluated the colony formation of CRC cells under different concentrations of oxaliplatin or lobaplatin administration, and we found that colony formation gradually decreased with increasing concentrations of oxaliplatin or lobaplatin (Fig. 1E–J). In the subsequent experiments, we used oxaliplatin (30 μM) or lobaplatin (16 μg/ml) to treat CRC cells. By western blot assay, we observed that oxaliplatin and lobaplatin activated the expression of Nrf2 and HO-1 in CRC cells (Fig. 1K,L). Furthermore, we extracted nuclear protein and the results showed that the Nrf2 expression of the nuclear protein was high with drug treatment.

### 4-OI, an Nrf2 activator, inhibited apoptosis by reducing ROS production and attenuated the cytotoxic effects of oxaliplatin and lobaplatin on CRC cells.

**Figure 2:**
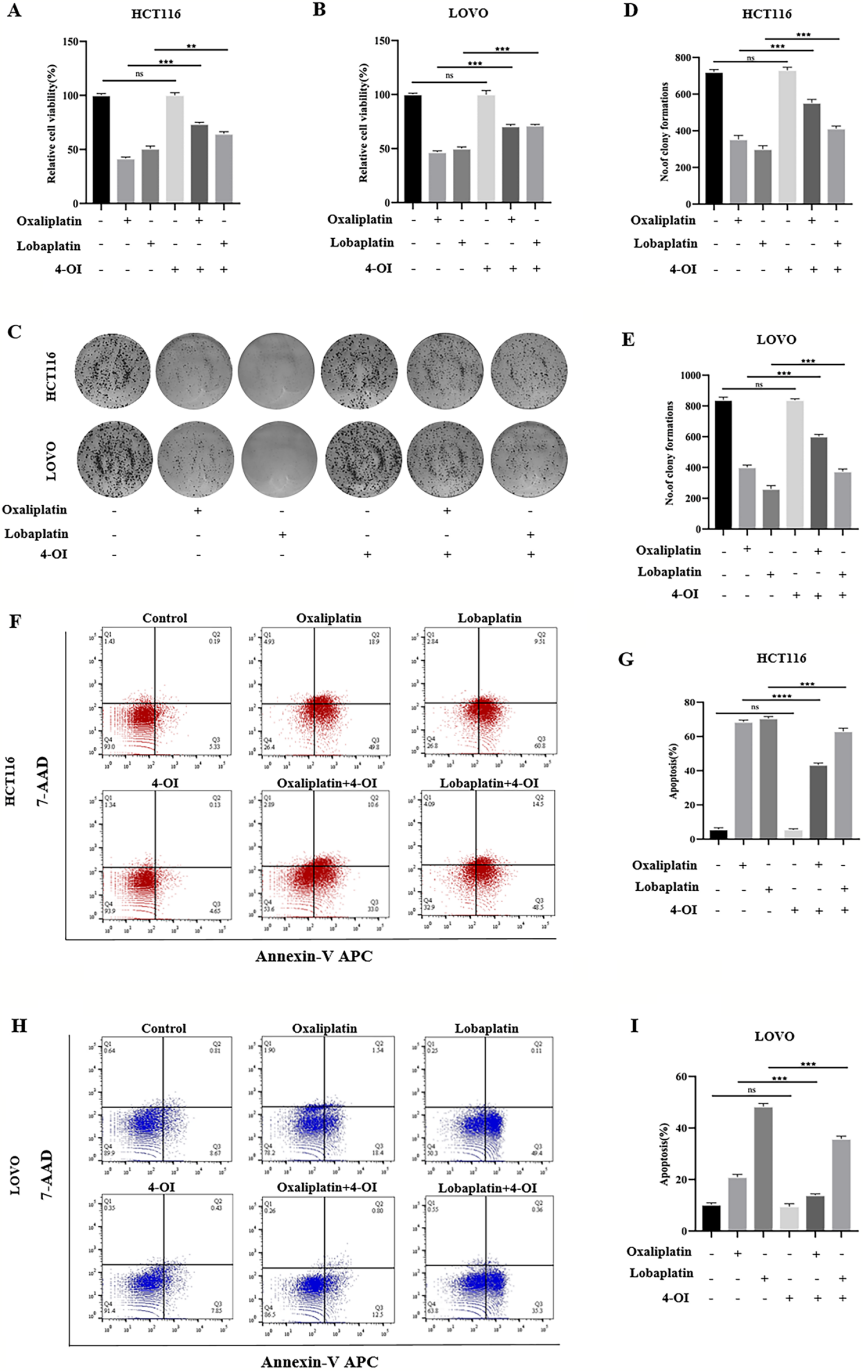
4-OI attenuated the cytotoxic effects of oxaliplatin and lobaplatin on CRC cells. (**A**, **B**) 4-OI weakened the inhibitory effects of oxaliplatin and lobaplatin on the viability of CRC cells by the MTS assay. (**C**–**E**) Compared with oxaliplatin or lobaplatin alone, 4-OI combined with oxaliplatin or lobaplatin increased colony formation of CRC cells. (**F**–**I**) 4-OI inhibited oxaliplatin-induced and lobaplatin-induced apoptosis by flow cytometry. Data are shown as the mean ± SD, **p < 0.01; ***p < 0.001; ns (not significant).

**Figure 3:**
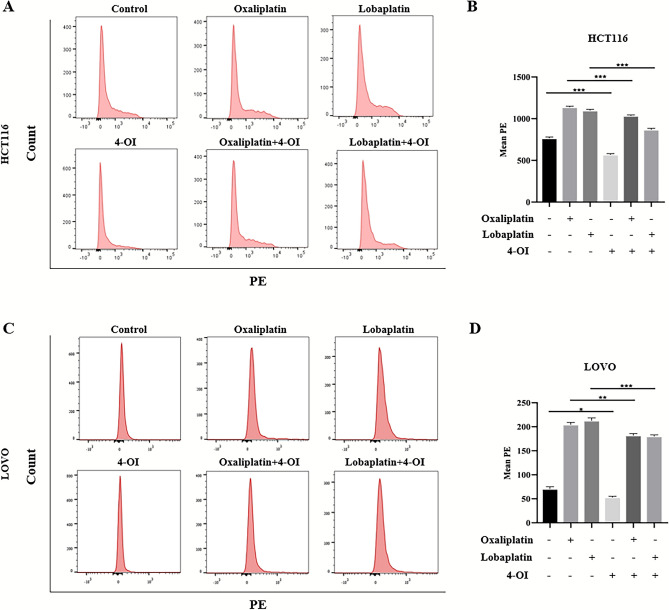
4-OI reduced oxaliplatin-associated and lobaplatin-associated ROS production. (**A**–**D**) Compared with the application of oxaliplatin or lobaplatin alone, 4-OI combined with oxaliplatin or lobaplatin significantly reduced the ROS production by flow cytometry. Data are shown as the mean ± SD, *p < 0.05; **p < 0.01; ***p < 0.001.

Based on the previous studies, we found that platinum-based drugs increased the expression of Nrf2 in CRC cells. Studies have found that the up-regulation of Nrf2 expression promoted the survival of tumor cells. Thus, we hypothesized whether CRC cells were resistant to platinum-based drugs through the overexpression of Nrf2. Studies have confirmed that the 4-OI is one of the Nrf2 activators^26–28^. To verify our hypothesis, we applied 4-OI to activate Nrf2 and observed the effect of 4-OI (200 μM) combined with oxaliplatin(30 μM) or lobaplatin(16 μg/ml) on CRC cells. We observed that 4-OI combined with oxaliplatin or lobaplatin increased the viability of CRC compared with oxaliplatin or lobaplatin alone by MTS assay (Fig. 2A,B). Also, 4-OI combined with oxaliplatin or lobaplatin increased colony formation of CRC cells compared with oxaliplatin or lobaplatin alone (Fig. 2C–E). The Annexin V/7-AAD double staining assay was used to detect the effect of 4-OI combined with oxaliplatin or lobaplatin on the apoptosis of CRC cells. We found that the combination of 4-OI with oxaliplatin or lobaplatin inhibited apoptosis in CRC cells (Fig. 2F–I). Excessive ROS production is one of the intrinsic pathways leading to apoptosis. To examine the effect of platinum-based drugs on ROS production in CRC cells after activation of Nrf2, flow cytometry was used to measure the ROS production in two CRC cell lines. We found that compared to the application of oxaliplatin or lobaplatin alone, 4-OI combined with oxaliplatin or lobaplatin decreased the production of ROS, which suggested that 4-OI may inhibit apoptosis by reducing ROS production (Fig. 3A–D). DMF, another clinically approved Nrf2 activator, attenuated the cytotoxic effects of oxaliplatin on CRC cells (Supplementary Fig. 1).

### The knockdown of Nrf2 promoted apoptosis by increasing ROS production and enhanced the cytotoxic effects of oxaliplatin and lobaplatin on CRC cells.

**Figure 4:**
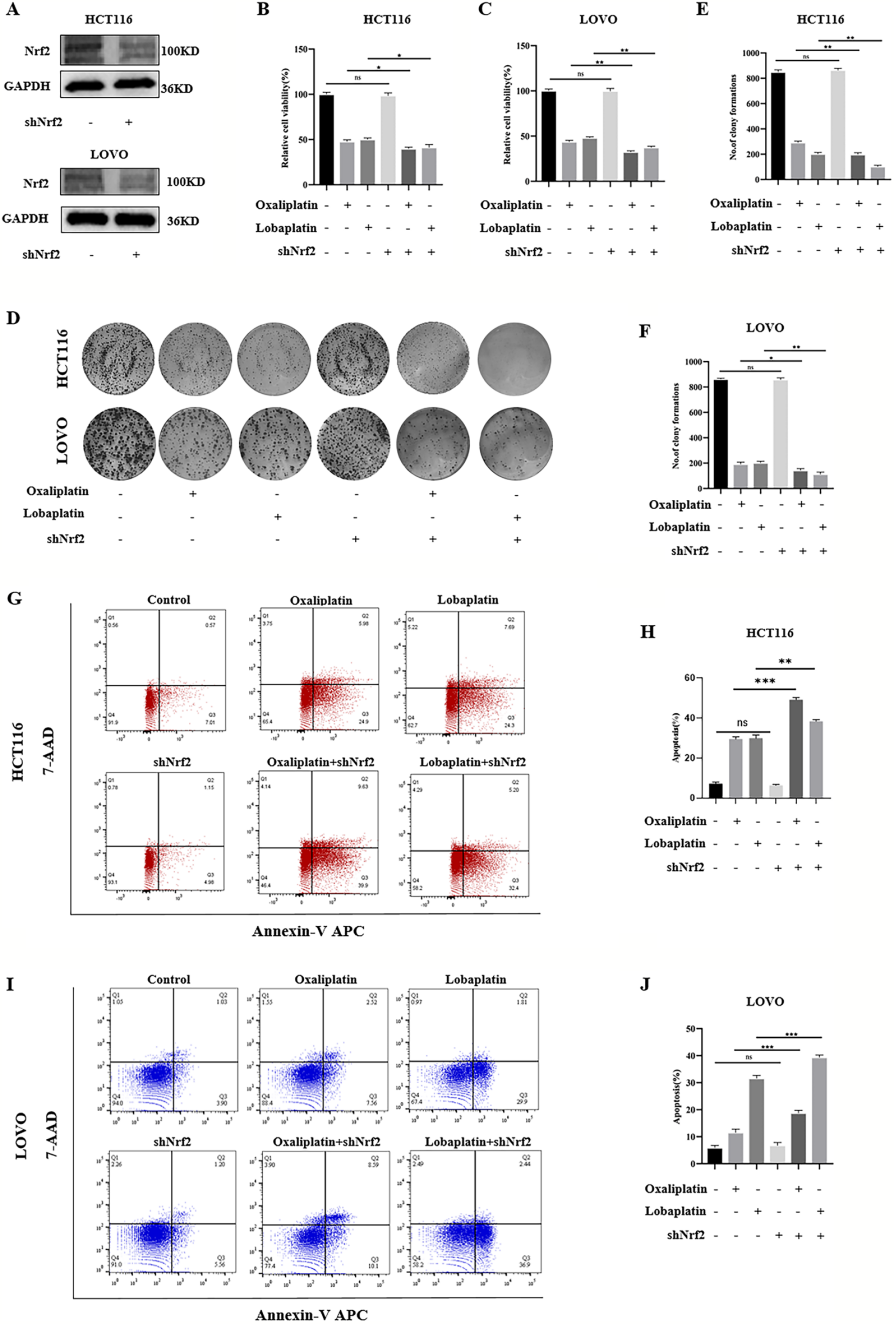
The knockdown of Nrf2 increased the cytotoxic effects of oxaliplatin and lobaplatin on CRC cells. (**A**) The stable knockdown of Nrf2 in HCT116 and LOVO cells after lentivirus transfection. (**B**, **C**) The knockdown of Nrf2 enhanced the inhibitory effects of oxaliplatin and lobaplatin on the viability of CRC cells by the MTS assay. (**D**–**F**) Compared to oxaliplatin or lobaplatin alone, the knockdown of Nrf2 combined with oxaliplatin or lobaplatin formed fewer colonies in CRC cells. (**G**–**J**) The knockdown of Nrf2 promoted oxaliplatin-induced and lobaplatin-induced apoptosis by flow cytometry. Data are shown as the mean ± SD, *p < 0.05; **p < 0.01; ***p < 0.001; ns (not significant).

**Figure 5:**
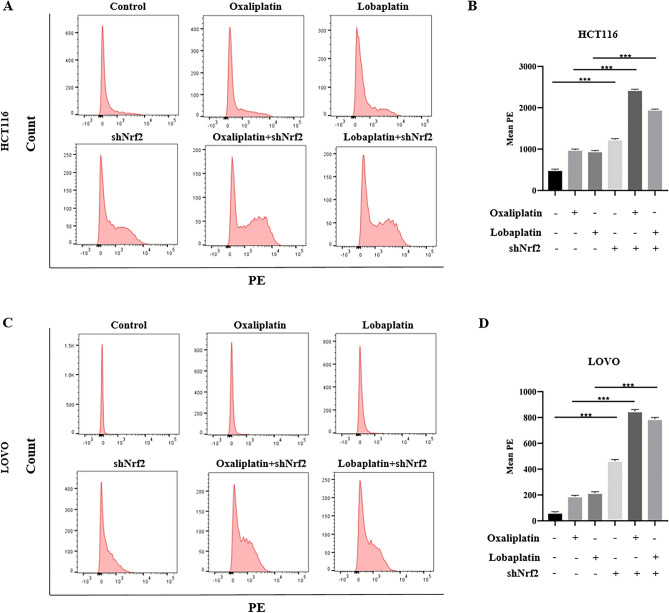
The knockdown of Nrf2 increased oxaliplatin-associated and lobaplatin-associated ROS production. (**A**–**D**) By the flow cytometry, compared to oxaliplatin or lobaplatin alone, the results showed that the knockdown of Nrf2 combined with oxaliplatin or lobaplatin increased the ROS production in CRC cells. Data are shown as the mean ± SD, ***p < 0.001.

Elevated expression of Nrf2 leads to reducing the sensitivity of platinum-based drugs on CRC cells. Will the sensitivity of platinum-based drugs on CRC cells increase if the expression of Nrf2 is reduced? To clarify this question, we used lentivirus to knock down Nrf2 in HCT116 and LOVO cells. The inhibition of Nrf2 was verified by western blot (Fig. 4A). Compared to oxaliplatin or lobaplatin alone, the MTS assay showed that the knockdown of Nrf2 combined with oxaliplatin or lobaplatin significantly decreased the viability of CRC cells (Fig. 4B,C). We observed similar results in the colony formation assay. Compared to oxaliplatin or lobaplatin alone, the knockdown of Nrf2 combined with oxaliplatin or lobaplatin formed fewer colonies in CRC cells (Fig. 4D–F). By the Annexin V/7-AAD double staining assay, the effect of apoptosis was detected. We found that the knockdown of Nrf2 combined with oxaliplatin or lobaplatin promoted apoptosis (Fig. 4G–J). Furthermore, the flow cytometry showed that the knockdown of Nrf2 combined with oxaliplatin or lobaplatin increased the ROS production in CRC cells (Fig. 5A–D).

### ML385, an Nrf2 inhibitor, enhanced the cytotoxic effects of oxaliplatin and lobaplatin on CRC cells.

**Figure 6:**
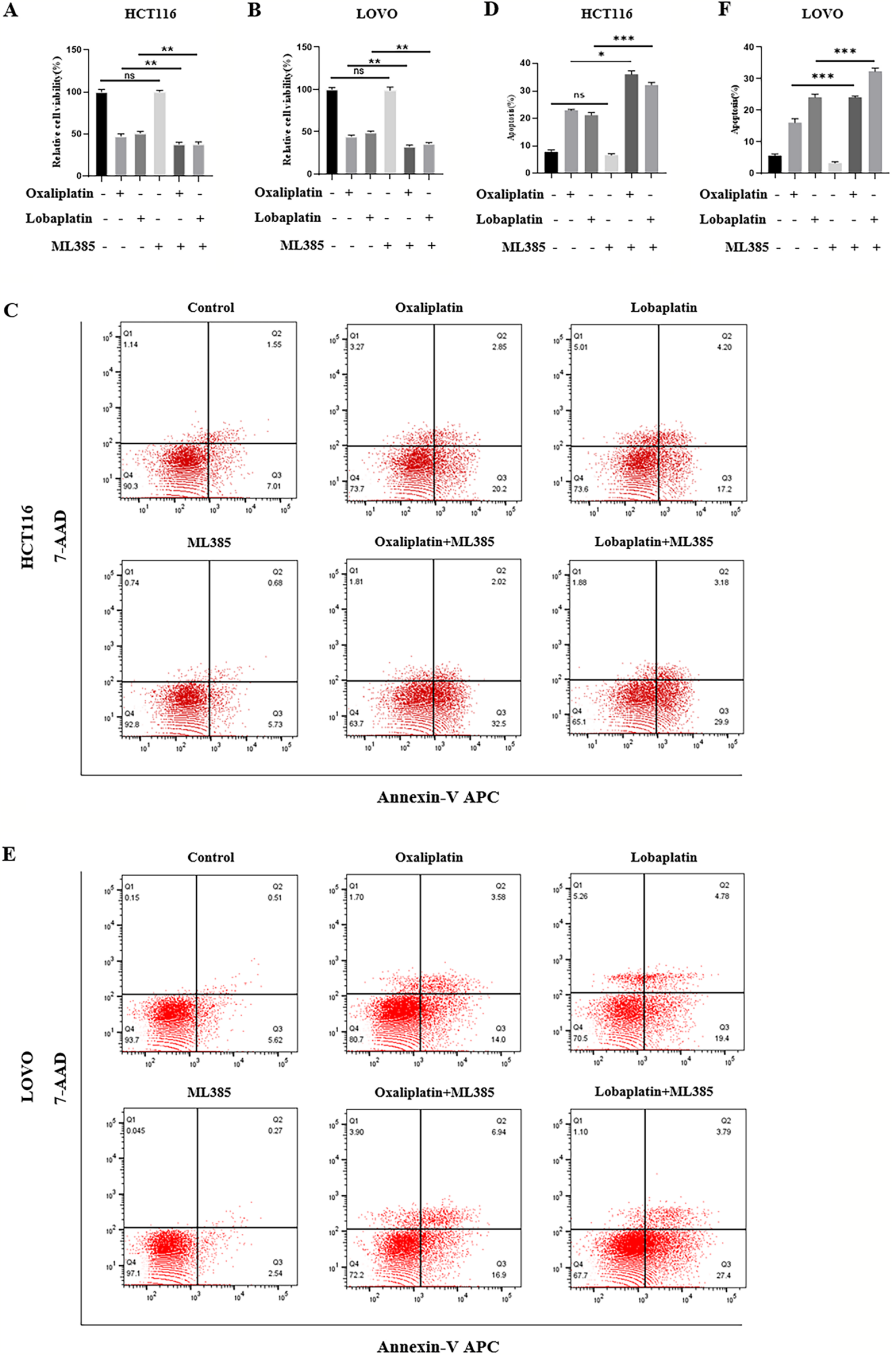
ML385 enhanced the cytotoxic effects of oxaliplatin and lobaplatin on CRC cells. (**A**–**B**) ML385 combined with oxaliplatin or lobaplatin significantly decreased the viability of CRC cells. (**C**–**F**) Compared to oxaliplatin or lobaplatin alone, the ML385 combined with oxaliplatin or lobaplatin promoted CRC cell apoptosis.

Then, we used ML385 to inhibit the transcriptional activity of Nrf2, and the results showed that ML385 combined with oxaliplatin or lobaplatin significantly decreased the viability of CRC cells (Fig. 6A,B). Compared to oxaliplatin or lobaplatin alone, the ML385 combined with oxaliplatin or lobaplatin promoted CRC cell apoptosis (Fig. 6C–F).

### The knockdown of Nrf2 caused 4-OI to lose the inhibitory effect of apoptosis and inactivate the inhibitory effect of 4-OI on CRC cell death.

**Figure 7:**
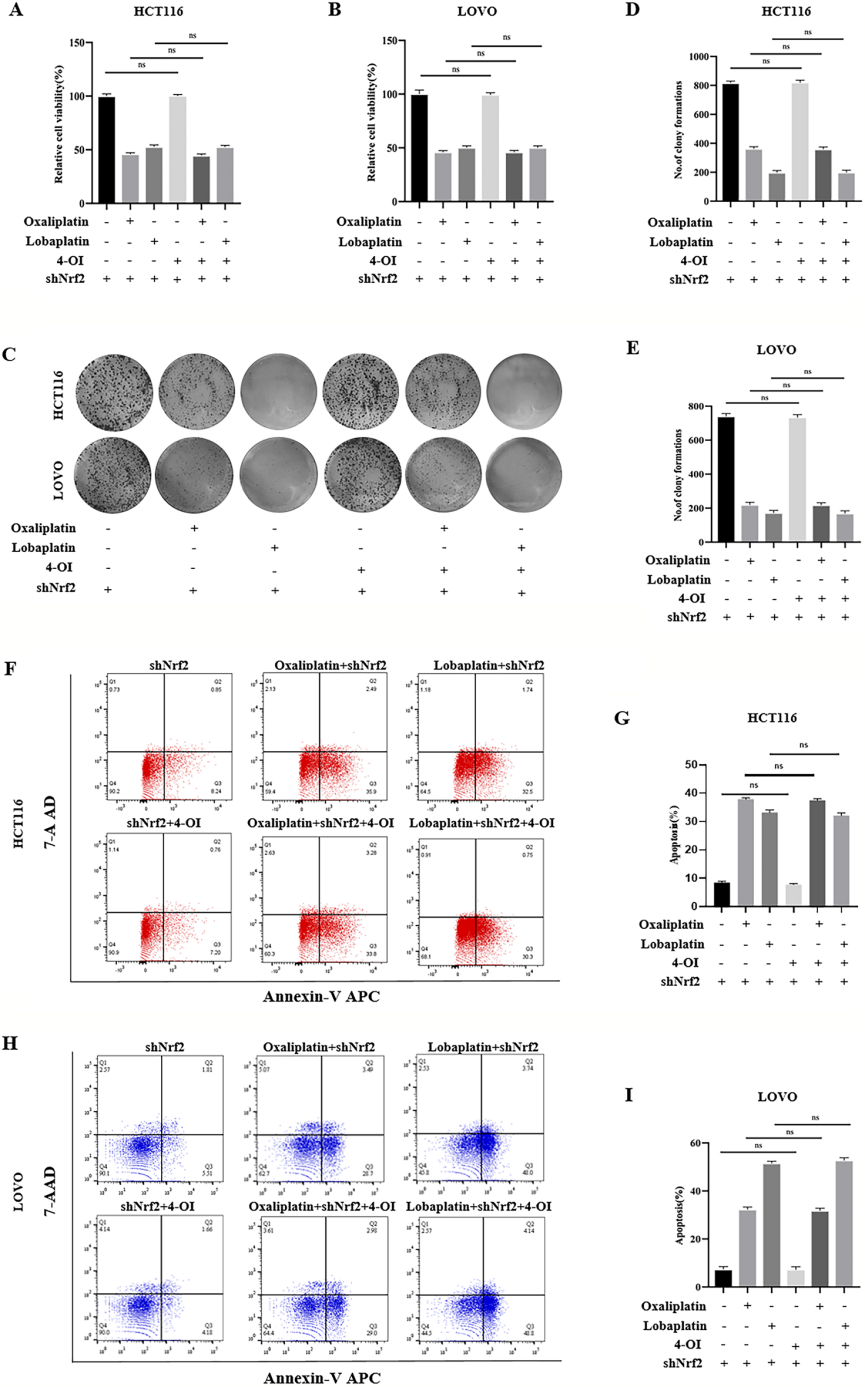
The knockdown of Nrf2 inactivated the inhibitory effect of 4-OI on CRC cell death. (**A**, **B**) Compared with oxaliplatin or lobaplatin alone, 4-OI combined with oxaliplatin or lobaplatin had no difference in the viability of the Nrf2 knockdown of CRC cells. (**C**–**E**) In the colony formation assay, compared with oxaliplatin or lobaplatin alone, 4-OI combined with oxaliplatin or lobaplatin had no difference in the colony formation of the Nrf2 knockdown of CRC cells. (**F**–**I**) 4-OI combined with oxaliplatin or lobaplatin had no difference in apoptosis of the Nrf2 knockdown of CRC cells compared with oxaliplatin or lobaplatin alone. Data are shown as the mean ± SD, ns (not significant).

In the above experiments, 4-OI increased the cytotoxic effects of oxaliplatin and lobaplatin on CRC cells, while the knockdown of Nrf2 showed the opposite effect. Therefore, we further investigated whether 4-OI acts by affecting Nrf2. In the MTS assay, compared with oxaliplatin or lobaplatin alone, we observed that 4-OI combined with oxaliplatin or lobaplatin had no difference in the viability of CRC cells of the Nrf2 knockdown (Fig. 7A,B). In the colony formation assay, we observed that 4-OI combined with oxaliplatin or lobaplatin had no difference in the colony formation of CRC cells of the Nrf2 knockdown (Fig. 7C–E). By the Annexin V/7-AAD double staining assay, the effect of 4-OI combined with oxaliplatin or lobaplatin on the apoptosis of CRC cells was detected. We found that 4-OI combined with oxaliplatin or lobaplatin had no difference in apoptosis of CRC cells of the Nrf2 knockdown compared with oxaliplatin or lobaplatin alone (Fig. 7F–I).

### The knockdown of Nrf2 enhanced the sensitivity of CRC cells to oxaliplatin-induced ferroptosis.

**Figure 8:**
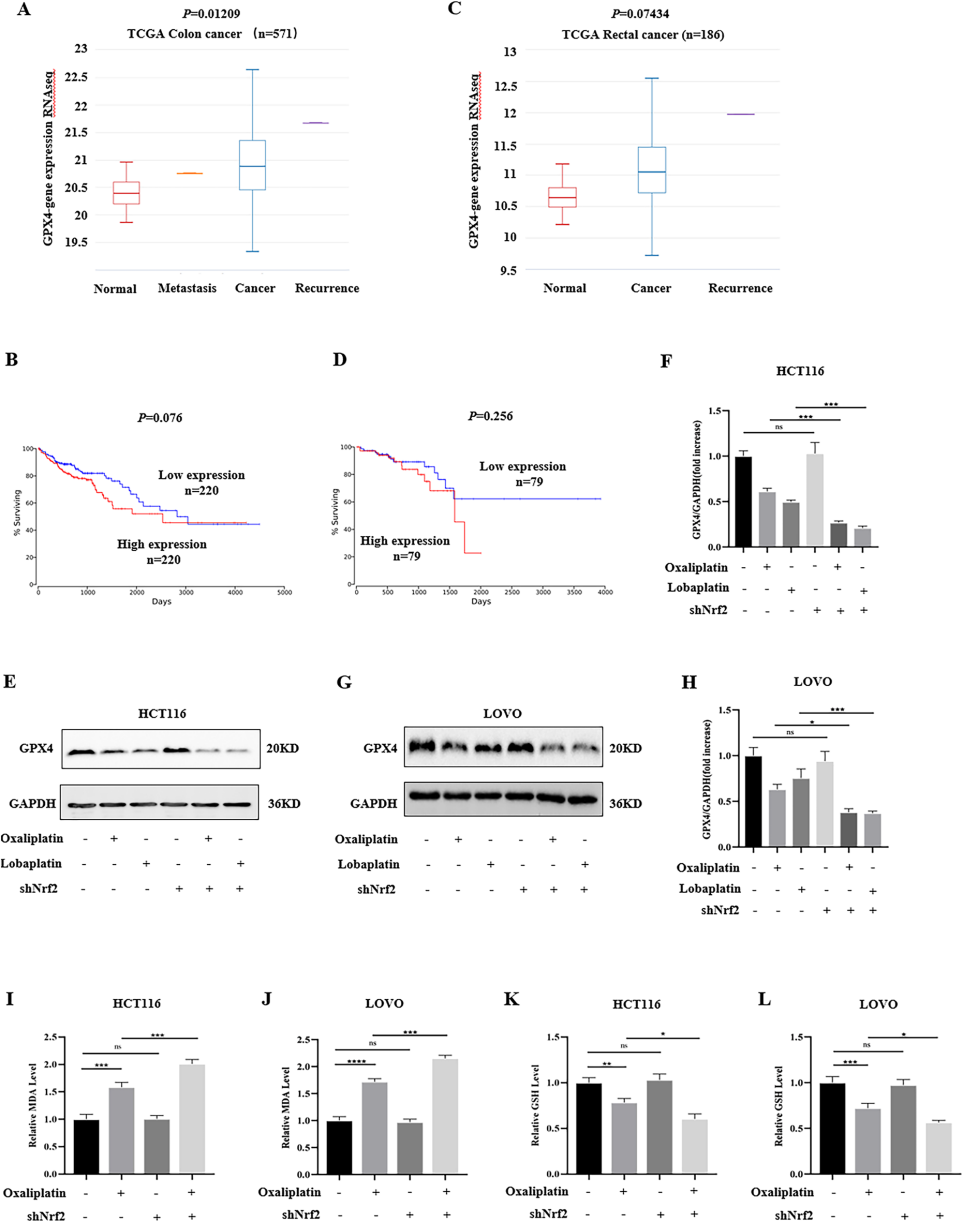
The knockdown of Nrf2 promoted oxaliplatin-induced ferroptosis in vitro. (**A**) The levels of GPX4 expression were increased in colon cancer tissues compared with normal tissues. (**B**) The survival curve showed a decreasing trend in the survival rate of colon cancer patients in the high GPX4 expression group. (**C**) The levels of GPX4 expression were increased in rectal cancer tissues compared with normal tissues. (**D**) The survival curve showed a decreasing trend in the survival rate of rectal cancer patients in the high GPX4 expression group. (**E**–**H**) By western blot, we found that oxaliplatin and lobaplatin inhibited the expression of GPX4. Compared with the control group, after knocking down Nrf2, oxaliplatin and lobaplatin reduced the expression of GPX4. (**I**, **J**) Oxaliplatin increased the MDA content. The knockdown of Nrf2 enhanced oxaliplatin to increase the MDA content. (**K**, **L**) Oxaliplatin decreased the GSH content. The knockdown of Nrf2 promoted oxaliplatin to reduce the GSH content. Data are shown as the mean ± SD, *p < 0.05; **p < 0.01; ***p < 0.001; ns (not significant).

Studies have reported that GPX4 is overexpressed in a variety of tumors and negatively correlated with the prognosis of patients. Through the public database, we analyzed the difference of GPX4 expression between CRC tissues and normal tissues and analyzed the effect of high and low GPX4 expression on the survival rate of CRC patients. Compared to normal tissues, we found that the expression of GPX4 was higher in colon cancer tissues (Fig. 8A). Meanwhile, compared to the low GPX4 expression group, the survival curve showed that the survival rate of the high GPX4 expression group had a downward trend (Fig. 8B). The expression of GPX4 in rectal cancer tissues was higher than that in normal tissues (Fig. 8C), and the survival rate of the high GPX4 expression group showed a downward trend (Fig. 8D). GPX4 can be used as a reference marker for judging cell ferroptosis. To clarify the effect of platinum-based drugs and the knockdown of Nrf2 on the expression of GPX4, we detected the expression of GPX4. We found that oxaliplatin and lobaplatin decreased the expression of GPX4. Compared with the control group, after knocking down Nrf2, oxaliplatin and lobaplatin decreased the expression of GPX4. The knockdown of Nrf2 enhanced the effects of oxaliplatin and lobaplatin to reduce the expression of GPX4 (Fig. 8E–H). MDA is one of indicator for reflecting lipid peroxidation damage. Oxaliplatin increased the MDA content, and the knockdown of Nrf2 enhanced oxaliplatin to increase the MDA content (Fig. 8I,J). As an antioxidant, GSH plays an important role in the body, and the depletion of GSH is considered as the essence of ferroptosis. Oxaliplatin reduced the GSH content, and the knockdown of Nrf2 promoted oxaliplatin to reduce the GSH content (Fig. 8K,L).

### The knockdown of Nrf2 enhanced the sensitivity of CRC cells to oxaliplatin-induced pyroptosis.

**Figure 9:**
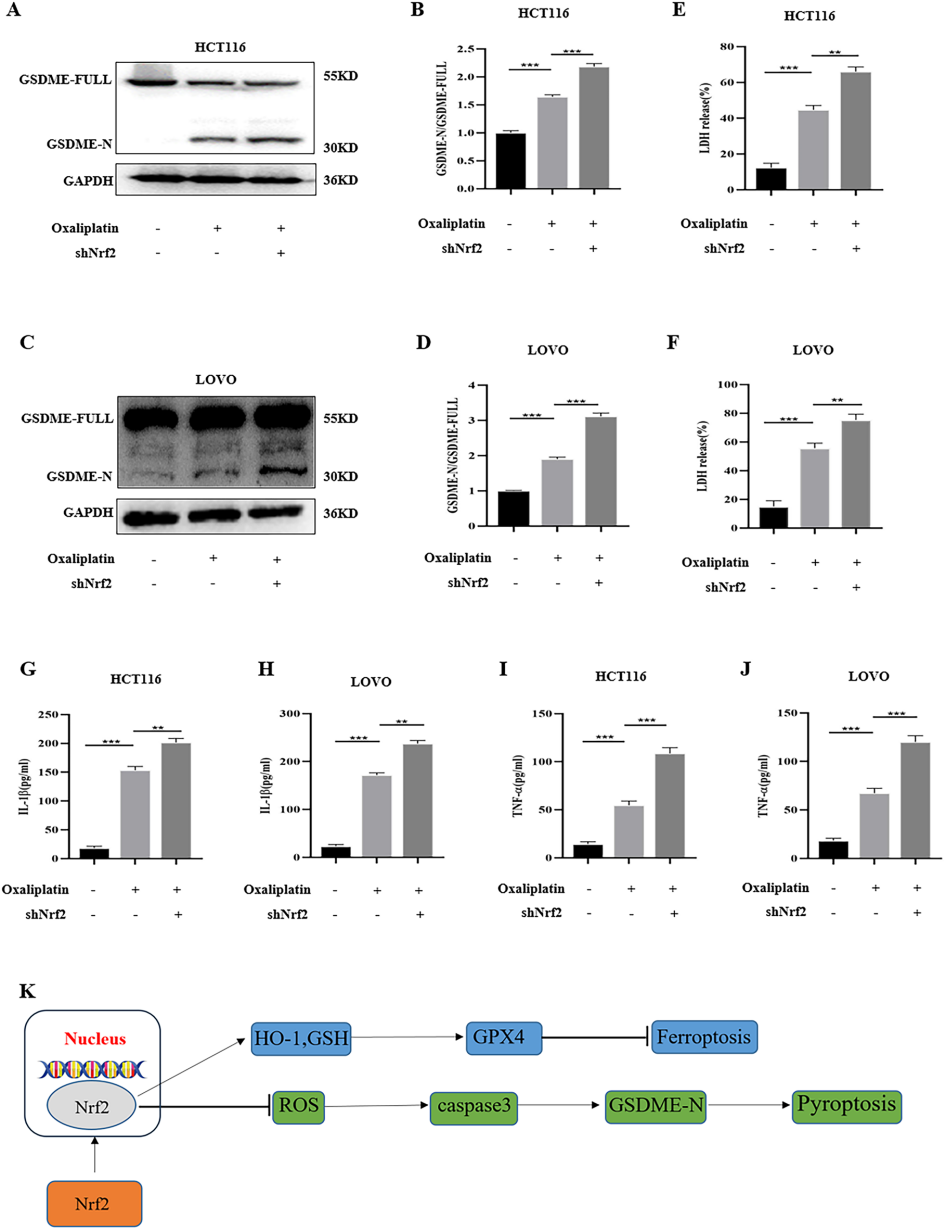
The knockdown of Nrf2 promoted oxaliplatin-induced pyroptosis in vitro. (**A**–**D**) By western blot, the results revealed that oxaliplatin raised the expression of GSDME-N. Compared with the control group, after knocking down Nrf2, oxaliplatin increased the expression of GSDME-N. (**E–J**) The knockdown of Nrf2 enhanced the increase IL-1β, TNF-α, and LDH release caused by oxaliplatin. (**K**)The schematic installation. Data are shown as the mean ± SD, **p < 0.01; ***p < 0.001.

GSDME contains two domains, gasdermin-N and gasdermin-C. GSDME-N has cytotoxic effects on cells and drives cells to pyroptosis. Research showed that the occurrence of pyroptosis can promote tumor cell death, which is beneficial to anti-tumor therapy. Therefore, we extracted GSDME by western blot. We found that oxaliplatin increased the expression of GSDME-N. When Nrf2 was knocked down, the expression of GSDME-N increased (Fig. 9A–D). Subsequently, IL-1β and TNF-α levels were detected by ELISA kit, and LDH release was detected by LDH assay kit. The results revealed that compared with the control group, oxaliplatin treatment increased IL-1β, TNF-α levels, and LDH release. The knockdown of Nrf2 enhanced the IL-1β, TNF-α, and LDH release caused by oxaliplatin. (Fig. 9E–J). The schematic installation was shown in Fig. 9K.

### The knockdown of Nrf2 enhanced the inhibition of oxaliplatin on HCT116 xenograft tumor growth in vivo.

**Figure 10:**
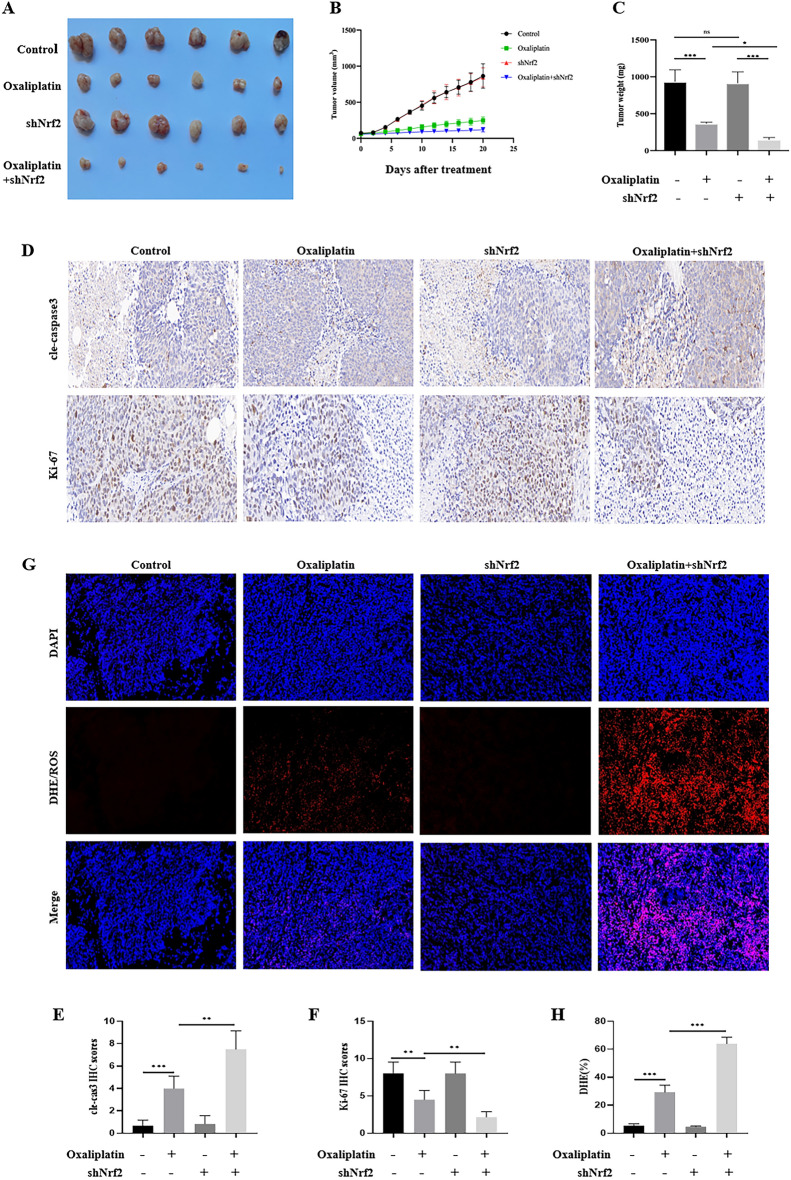
The knockdown of Nrf2 enhanced oxaliplatin-induced growth inhibition of HCT116 xenografts tumor in vivo. (**A–C**) The knockdown of Nrf2 enhanced the growth inhibition of oxaliplatin, and the tumors were significantly decreased in size, volume, and weight. (**D–F**) By the IHC staining of tumor tissues, in the Nrf2 knockdown + oxaliplatin group, we found that the expression of Ki-67 was diminished. In contrast, the expression of cle-caspase3 was relatively low in the Nrf2 knockdown + oxaliplatin group. (**G**, **H**) The knockdown of Nrf2 increased oxaliplatin-associated ROS production. Data are shown as the mean ± SD, *p < 0.05; ***p < 0.001; ns (not significant).

Based on the above results in vitro, we tested the effects of oxaliplatin and Nrf2 on HCT116 xenograft tumor growth in vivo. We found that in the control group and the Nrf2 knockdown group, the knockdown of Nrf2 did not change the growth of xenograft tumors. After oxaliplatin treatment, the growth of the xenograft tumor was inhibited. In the Nrf2 knockdown + oxaliplatin group, the inhibition of xenograft tumor was most obvious, and tumor size, volume, and weight were significantly reduced (Fig. 10A–C). Cle-caspase3 is a key mediator of apoptosis and reflects the level of apoptosis. The expression of Ki-67 can indicate the active degree of cell proliferation. The higher the expression of Ki-67, the more active the cell proliferation. The expressions of cle-caspase3 and Ki-67 in tumor tissue were detected by IHC. The results revealed that the expression of cle-caspase3 was low in the control group and the Nrf2 knockdown group, and the expression of cle-caspase3 was significantly increased in the Nrf2 knockdown + oxaliplatin group. In contrast, the expression of Ki-67 was high in the control group and the Nrf2 knockdown group, and the expression of Ki-67 was decreased in the Nrf2 knockdown + oxaliplatin group (Fig. 10D–F). DHE is a commonly used fluorescent detection probe for superoxide anion, which can freely permeate through living cells and enter into cells, and be oxidized by intracellular ROS to generate red fluorescence. According to the generation of red fluorescence in cells, the amount of ROS in cells can be judged. The results showed that the ROS production was low in the control group and the Nrf2 knockdown group, and the ROS production was significantly increased in the Nrf2 knockdown + oxaliplatin group than oxaliplatin group (Fig. 10G,H).

## Discussion

Chemotherapy is an important means of postoperative treatment for patients with advanced CRC, which can effectively prolong the survival time of patients, but the drug resistance of tumor cells is the main reason for the failure of chemotherapy^29^. The occurrence and development of drug resistance is an extremely complex process, which includes changes in the microenvironment and cellular genetic material^30^. At present, the specific mechanism of chemoresistance in CRC is not fully understood, mainly involving enhanced DNA damage repair, abnormal expression and function of transporter family, characteristics of tumor stem cells and abnormal activation of signal transduction pathways. Currently, oxaliplatin-containing chemotherapy is the first-line regimen for postoperative chemotherapy for advanced CRC, and oxaliplatin resistance will seriously affect the prognosis^31^. Therefore, there is an urgent need to improve sensitivity to chemotherapy and carry out individualized treatment.

Nrf2 has been recognized as a central hub to restore cellular redox balance. Under basal conditions, Nrf2 activity is tightly restricted by binding with Keap1 protein in the cytoplasm. Studies have shown that overactivation of Nrf2 is thought to be an intermediate link in cell proliferation and is involved in cancer resistance to treatment^32^. In addition, Nrf2, as a major antioxidant regulator, is involved in ROS detoxification and can reduce the damage to cells induced by chemotherapeutic drugs^33^. In our study, we found that both oxaliplatin and lobaplatin treatment of colorectal cancer cells elevated Nrf2 expression to counteract the effects of chemotherapeutic drugs. However, Liu et al.^34^ reported oxaliplatin suppresses the Nrf2 signaling pathway. Different concentrations of oxaliplatin may explain this reason, and oxaliplatin inhibits Nrf2 signaling pathway at low concentrations and promotes Nrf2 at high concentrations. We then used lentivirus to knock down Nrf2 in HCT116 and LOVO cell lines, and the results showed that intracellular ROS levels were significantly increased. Cell viability experiments and plate cloning experiments revealed that the cell viability in the Nrf2 knockdown group was significantly lower than that in the control group when they received the same concentration of oxaliplatin or lobaplatin. By flow cytometry, we found that the apoptosis ratio of HCT116 and LOVO cells in the knockdown group was significantly increased in the knockdown group treated with chemotherapy drugs. These results show that Nrf2 knockdown can significantly enhance the antitumor effect of chemotherapeutic drugs. Garufi et al.^35^ also reported that inhibiting Nrf2 was a useful strategy for anticancer chemotherapy. Another study also showed that inhibiting Nrf2 signaling in colon cancer patients with Her2 overexpression was an important strategy to overcome oxaliplatin resistance, which further indicating Nrf2 was an important target for enhancing chemotherapy sensitivity^36^. To further validate our conclusions, we used 4-OI, an Nrf2 activator. The results showed that 4-OI promoted tumor cell growth, and reduced the anti-tumor effect of oxaliplatin and lobaplatin. The reason may be that 4-OI activates Nrf2, reduces ROS production, and increases chemoresistance. Finally, we used HCT116 cells to conduct tumorigenesis experiments in mice, and the results revealed that the tumor volume of the mice in the Nrf2 knockdown group was significantly reduced after oxaliplatin treatment.

Ferroptosis is a new form of programmed cell death, which features iron-dependent lipid peroxidation^37,38^. During ferroptosis, free intracellular Fe^2+^ reacting with H_2_O_2_ to generate Fe^3+^ induces lipid peroxidation of polyunsaturated fatty acids, which ultimately mediates oxidative damage to biofilms and promotes cell death^39^. Subsequently, cystine can be converted to cysteine for the synthesis of the endogenous antioxidant glutathione (GSH)^38,40,41^. Finally, GPX4 utilizes GSH to scavenge excess intracellular ROS and reduce the content of toxic lipid peroxides^42^. Studies have shown that activation of ferroptosis effectively prevents tumor progression and enhances the efficacy of chemotherapy^43–45^. For example, Yang et al.^46^ reported that cetuximab promoted ferroptosis in KRAS mutant colorectal cancer to inhibit tumor growth. Wang et al.^47^ reported GSTZ1 sensitized hepatocellular carcinoma cells to sorafenib-induced ferroptosis. GPX4 can be used as one of the indicators for judging ferroptosis, and the inactivation of GPX4 is an important step in ferroptosis^48^. Studies have shown that compared to normal tissues, the expression level of GPX4 in tumor tissues is significantly increased^49,50^. We also found the expression of GPX4 in colon cancer tissues was higher than that in normal tissues through public database analysis. Meanwhile, both oxaliplatin and lobaplatin treatment reduced the expression of GPX4 and promote the occurrence of ferroptosis in vitro experiments. As an important scavenger of ROS, Nrf2 can promote the expression of GPX4 and reduce ferroptosis. We found that after the Nrf2 knockdown group received chemotherapy, the expression of GPX4 was significantly decreased, the expressions of GSH and MDA in the cells were significantly decreased, and the proportion of apoptosis was significantly increased. Next, we stained the tumor of the mice with DHE/ROS, and the results showed that the expression of ROS in the tumor of the knockdown group was significantly increased. These results suggested that Nrf2 knockdown promoted ferroptosis, which in turn enhanced the antitumor effect of chemotherapeutics.

In recent years, more and more studies have shown that GSDME-mediated pyroptosis has a good anti-tumor effect. During tumor formation, GSDME is in a state of transcriptional repression through methylation modification. When exposed to different degrees of external stimuli, such as tumor necrosis factor or chemotherapeutic drugs, it can activate caspase3 and activate GSDEM, resulting in cell pyroptosis. Therefore, GSDME-mediated pyroptosis may play an important role in chemotherapeutic drug-induced cytotoxicity. Zhang et al.^51^ reported that chemotherapeutic paclitaxel and cisplatin induced pyroptosis via caspase-3/GSDME activation in lung cancer cells. Another study showed that miltirone induces hepatocellular carcinoma cell death by GSDME-dependent pyroptosis^52^. Meanwhile, a study published in *Nature* revealed GSDME suppressed tumor growth by enhancing anti-tumor immunity^53^. In this study, we also found that the expression of GSDME-N in colon cancer cells treated with oxaliplatin was significantly increased, and the release of LDH and the content of TNF-α in the supernatant were significantly increased. As an important anti-inflammatory and antioxidant molecule, Nrf2 can inhibit the production of TNF-α, which may further inhibit GSDME activation. To test our hypothesis, we treated Nrf2 knockdown cells and control cells with drugs, respectively, and the results revealed that the knockdown group cell had higher GSDME-N expression and higher LDH release. These results showed that Nrf2 inhibition enhanced chemotherapeutic drug-induced pyroptosis and improved chemosensitivity of colorectal cells.

In summary, our study showed that Nrf2 inhibition increases sensitivity to chemotherapy of colorectal cancer. Mechanistically, the effect caused by Nrf2 inhibition was closely related to the promotion of ferroptosis and pyroptosis.

### Supplementary Information

Supplementary Figure 1.

Supplementary Legends.

Supplementary Information 1.

## Data Availability

The data used to support the findings of this study are available from the corresponding authors upon request.
