# Supplementary figures and images for: Nrf2 inhibition increases sensitivity to chemotherapy of colorectal cancer by promoting ferroptosis and pyroptosis
Source: Sci Rep. 2023 Sep 1;13:14359. doi: 10.1038/s41598-023-41490-x (PMC10474100; doi:10.1038/s41598-023-41490-x)

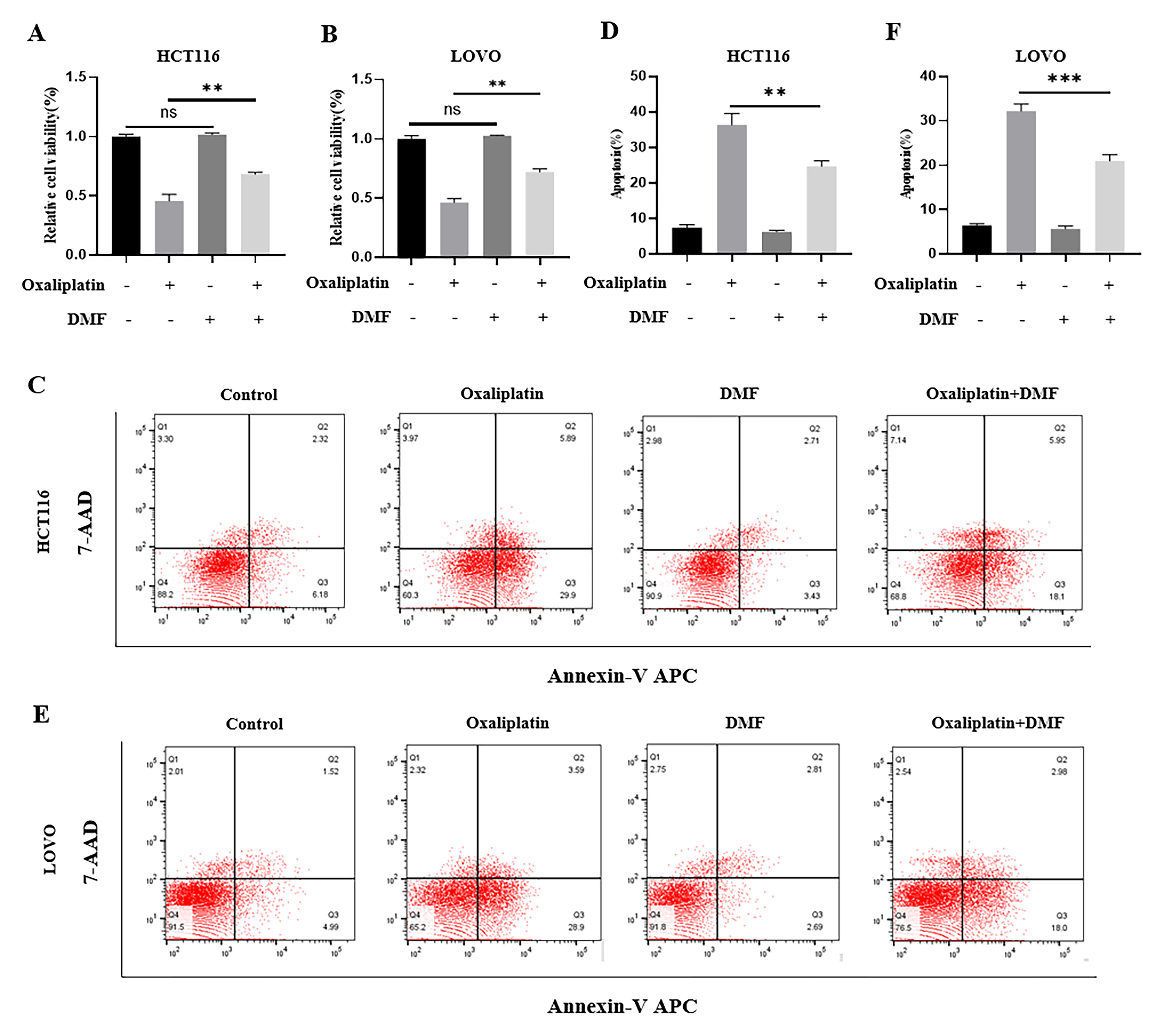

Supplement: Supplementary file 1 — Supplementary Figure 1. [file 41598_2023_41490_MOESM1_ESM.tif]
