# Supplementary material for: Nrf2 inhibition increases sensitivity to chemotherapy of colorectal cancer by promoting ferroptosis and pyroptosis
Source: Sci Rep. 2023 Sep 1;13:14359. doi: 10.1038/s41598-023-41490-x (PMC10474100; doi:10.1038/s41598-023-41490-x)
Supplement: Supplementary file 2 — Supplementary Legends. [file 41598_2023_41490_MOESM2_ESM.docx]

**Supplementary Figure legend**

**Supplementary Figure 1 DMF attenuated the cytotoxic effects of oxaliplatin on CRC cells. (A, B)** DMF weakened the inhibitory effects of oxaliplatin on the viability of CRC cells. **(C-F)** DMF inhibited oxaliplatin-induced apoptosis by flow cytometry on HCT116 and LOVO cells. Data are shown as the mean ± SD, **p < 0.01; ***p < 0.001; ns (not significant).
