# Supplementary material for: Nrf2 inhibition increases sensitivity to chemotherapy of colorectal cancer by promoting ferroptosis and pyroptosis
Source: Sci Rep. 2023 Sep 1;13:14359. doi: 10.1038/s41598-023-41490-x (PMC10474100; doi:10.1038/s41598-023-41490-x)

Figure 1K


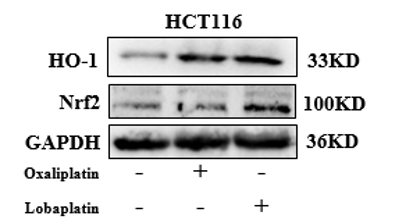


1. HCT116 Nrf2

1. HCT116 GAPDH







1. HCT116 HO-1


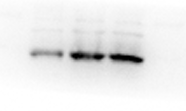


2. HCT116 Nrf2

2. HCT116 GAPDH


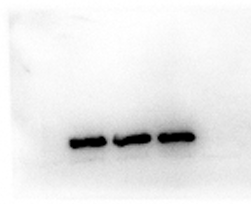

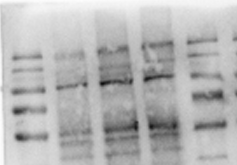


2. HCT116 HO-1


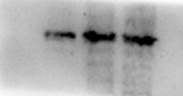


3. HCT116 Nrf2

3. HCT116 GAPDH


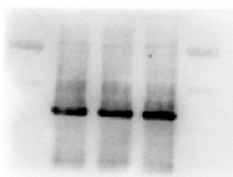

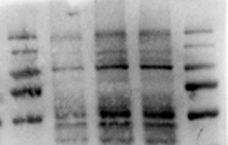


3. HCT116 HO-1


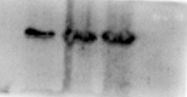


Figure 1K


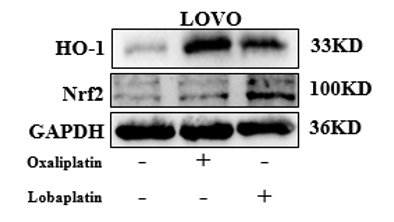


1. LOVO Nrf2

1. LOVO GAPDH


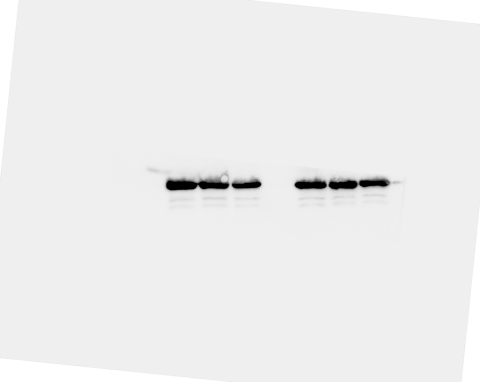

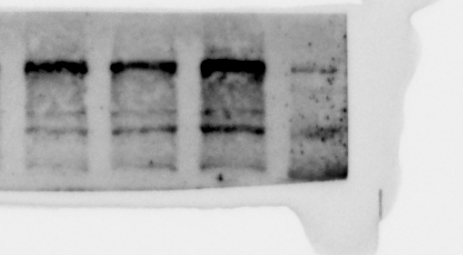


1. LOVO HO-1


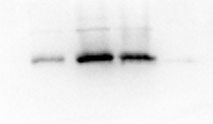


2. LOVO Nrf2

2. LOVO GAPDH


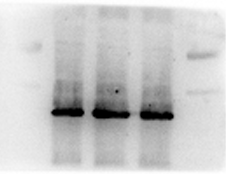

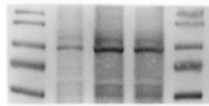


2. LOVO HO-1


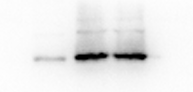


3. LOVO Nrf2

3. LOVO GAPDH


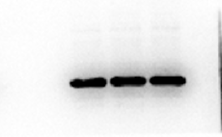

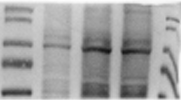


3. LOVO HO-1


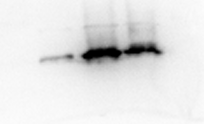


Figure 1K


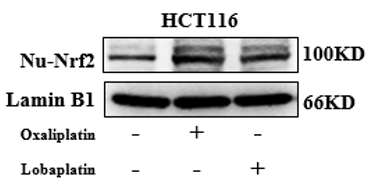


HCT116 Nu-Nrf2

HCT116 Lamin B1


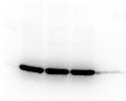

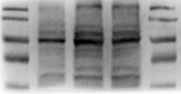


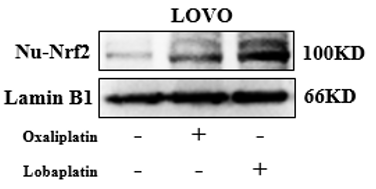


LOVO Nu-Nrf2

LOVO Lamin B1


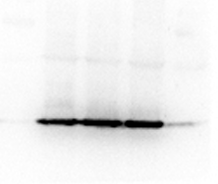

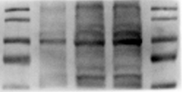


Figure 4A


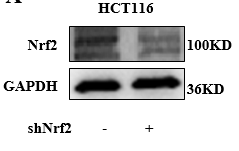


1. HCT116 Nrf2

1. HCT116 GAPDH


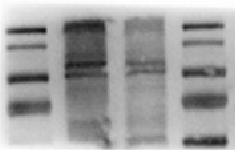

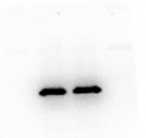


2. HCT116 Nrf2

2. HCT116 GAPDH


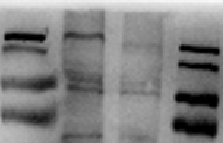

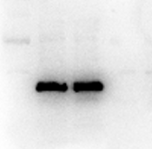


3. HCT116 Nrf2

3. HCT116 GAPDH


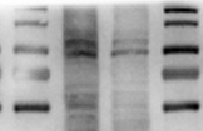


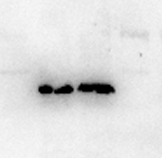


Figure 4A


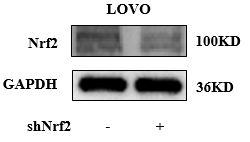


1. LOVO Nrf2

1. LOVO GAPDH


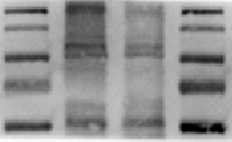


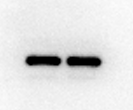


2. LOVO Nrf2

2. LOVO GAPDH


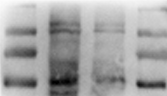

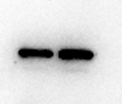


3. LOVO GAPDH

3. LOVO Nrf2


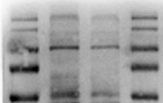

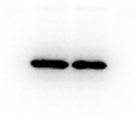


Figure 8E


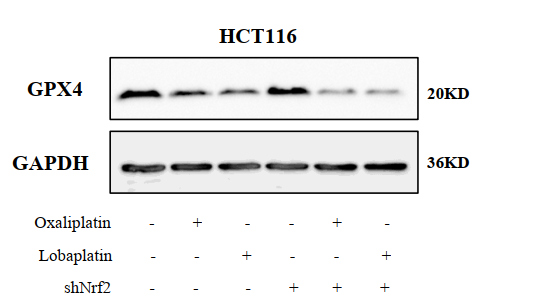


HCT116 GPX4

HCT116 GAPDH







Figure 8G


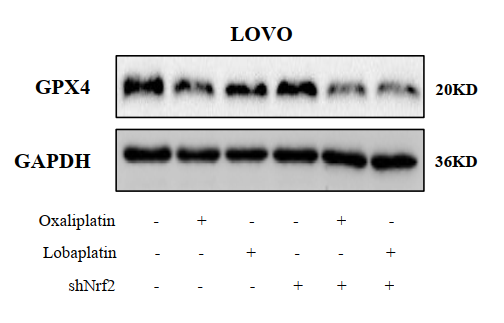


LOVO GPX4

LOVO GAPDH


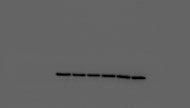




Figure 9A


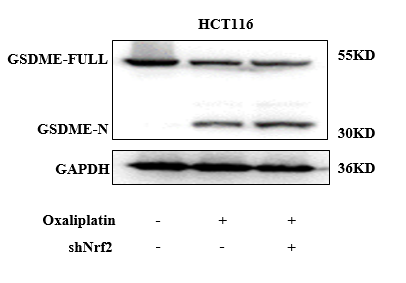


1. HCT116 GSDME

1. HCT116 GAPDH


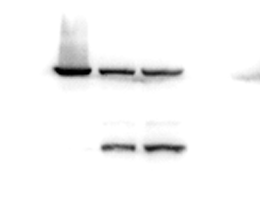


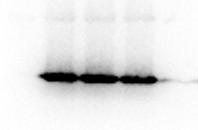


2. HCT116 GSDME

2. HCT116 GAPDH


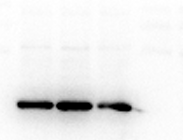

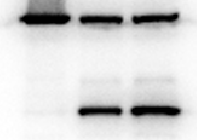


3. HCT116 GSDME

3. HCT116 GAPDH


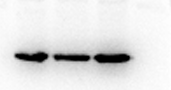

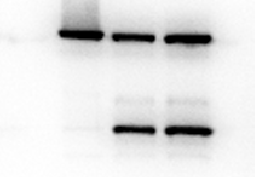


Figure 9C


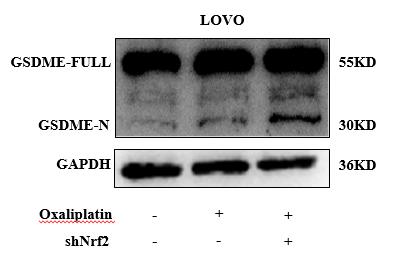


1. LOVO GSDME

1. LOVO GAPDH


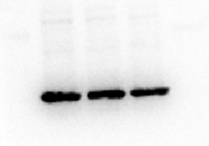

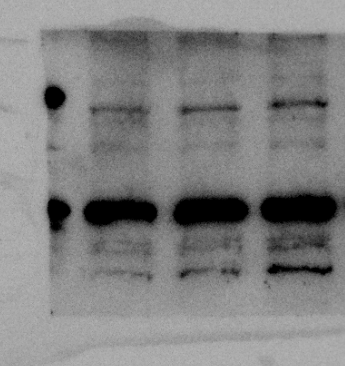


2. LOVO GSDME

2. LOVO GAPDH


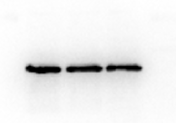

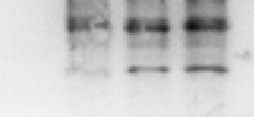


3. LOVO GSDME

3. LOVO GAPDH


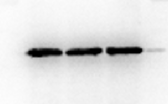

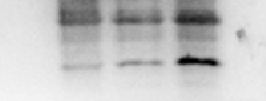

Supplement: Supplementary file 3 — Supplementary Information 1. [file 41598_2023_41490_MOESM3_ESM.docx]
